# Mechanisms of chemotherapy-induced human ovarian aging: double strand DNA breaks and microvascular compromise

**DOI:** 10.18632/aging.100363

**Published:** 2011-08-22

**Authors:** Reza Soleimani, Elke Heytens, Zbigniew Darzynkiewicz, Kutluk Oktay

**Affiliations:** ^1^ Laboratory of Molecular Reproduction, Institute for Fertility Preservation, Departments of Obstetrics & Gynecology and Cell Biology & Anatomy, New York Medical College, Valhalla, New York, USA; ^2^ Brander Cancer Research Institute and Department of Pathology, New York Medical College, Valhalla, New York, USA

**Keywords:** Ovary, Doxorubicin, Double Strand DNA Breaks, Apoptosis, Fertility Preservation, Aging, Chemotherapy

## Abstract

The mechanism of chemotherapy-induced acceleration of ovarian aging is not fully understood. We used doxorubicin, a widely used cancer chemotherapeutic, in a variety of *in vivo* xenograft, and *in vitro* models to investigate the impact of chemotherapy-induced aging on the human ovary. Doxorubicin caused massive double-strand-DNA-breaks in primordial follicles, oocytes, and granulosa cells in a dose dependent fashion as revealed by accumulating γH2AX foci. This damage was associated with apoptotic oocyte death and resulted in the activation of ATM. It appeared that the repair response enabled a minor proportion of oocytes (34.7%) and granulosa cells (12.1%) to survive while the majority succumbed to apoptotic death. Paradoxically, inhibition of ATM by KU-55933 resulted in improved survival, probably via prevention of downstream activation of TAp63α. Furthermore, doxorubicin caused vascular and stromal damage in the human ovary, which might impair ovarian function both pre- and post-menopausally. Chemotherapy-induced premature ovarian aging appears to result from a complex process involving both the germ- and non-germ cell components of the ovary. These effects may have clinical implications in aging both for premenopausal and postmenopausal cancer survivors.

## INTRODUCTION

Chemotherapy-induced ovarian failure not only increases the risk for early menopause-related complications but also results in infertility in young female cancer survivors [1, 2]. In general, underlying mechanisms of this chemotherapy-induced ovarian aging is inadequately characterized in humans.

The goal of this study was to explore the mechanism of the acceleration of human ovarian aging as a result of chemotherapy. To investigate this effect we utilized a commonly administered cancer chemotherapeutic, doxorubicin, in an *in vivo* xenograft as well as *in vitro* models, using a double-species approach. DNA damage response induced by doxorubicin was assessed immunocytochemically by detecting histone H2AX phosphorylation on Ser139 [3] and activation of Ataxia Telangiectasia Mutated protein kinase (ATM) through its phosphorylation on Ser1981, using the respective phospho-specific Abs [4]. The extensive nuclear labeling with these Abs, especially in form of characteristic foci, is considered to report the presence of DSBs, the potentially lethal DNA lesions.

## METHODS

This study was carried out in strict accordance with the recommendations in the Guide for the Care and Use of Laboratory Animals of the National Institutes of Health and the New York Medical College (NYMC) Institutional Animal Care and Use Committee. The study protocol was approved by the institutional review board of NYMC and written informed consent was obtained from all participants involved in the study. All surgery was performed under anesthesia, and all efforts were made to minimize suffering. Ovarian tissue fragments were obtained from consenting females patients, or their parents in the case of minors, (n=6, age range 2-37) during ovarian tissue cryopreservation procedures before chemotherapy.

The effect of doxorubicin was studied both with *in vitro* cultured human ovarian cortical tissue (OCT) and an *in vivo* human to severe combined immunodeficiency (NOG-SCID) (Taconic, USA) mice xenotransplantation model, as well as in *in vivo* mouse experiments.

### Human ovarian tissue culture

OCTs were cut into 1mm^3^ pieces. They were then randomly selected and cultured in multi-well plates (# 35-3078, Becton, Dickinson, NJ, USA) with MEM-alpha medium (Gibco, Invitrogen, Carlsbad, USA) supplemented with 10% bovine serum albumin (v/v)(Sigma-Aldrich, St. Louis, MO, USA) and 1% insulin-transferrin-selenium (Gibco, Invitrogen, Carlsbad, USA) containing 1, 10 and 100 μg/ml of doxorubicin (Sigma-Aldrich, St Louis, MO, USA) for 24-72h (n=8 in each experimental point). In controls, vehicle (sterile normal saline) was added to the culture medium in control group. Cultured OCT samples were recovered and processed for further histological evaluation.

### Human ovarian tissue xenografting

OCT pieces (1mm^3^) were selected randomly and transplanted into the dorsal muscles of seven-week-old SCID mice (NOG, Taconic, USA) as previously described (13-16) (n=8). Briefly, human ovarian fragments were collected in modified M199 medium (Gibco, Invitrogen, Carlsbad, USA). Recipient animals were anesthetized by ketamine 75 mg/kg i.p. (Ketathesia, OH, USA) and xylazine 1 mg/kg i.p. (AnaSed, Shanandoah, Iowa, USA) [5]. A small incision was made in the skin on the dorsal midline of the recipient animal, and OCT pieces (n=2/animal) were transplanted into the dorsal muscles in recipient mice by means of a fine watchmakers' forceps. Skin incision was closed under aseptic conditions. Animals were allowed to recover for ten days and 10 mg/kg doxorubicin was administered to the xenografted animals intravenously. In contrast, control animals were treated with vehicle. OCT grafts were recovered 24h later and analyzed for vascular density by CD31 expressing micro-vessels/mm^2^ field, percentage of primordial follicles with DSBs by γH2AX staining and those that are apoptotic by AC3 expression.

### *In vivo* assessment of the impact of doxorubicin in the mouse ovary

Due to the non-uniformity of human primordial follicles distribution in OCTs, the number of primordial follicles and apoptotic follicles densities were compared in ovaries of doxorubicin-treated SCID mice compared to vehicle-treated controls (n=6).

Furthermore, to study the *in vivo* effect of doxorubicin on ovarian follicles and their cumulus cells, mice (n=7) (B6D2 and FBV, Taconic, USA) were injected with a single dose of doxorubicin 10 mg/kg. Animals were sacrificed 24h later, ovaries were collected, small preantral follicles (100-120 μm in diameter) (n=140) and GV oocytes (n=94) were mechanically isolated by a fine needle (25 5/8-G; Becton-Dickinson NJ, USA) and fixed in 4% of methanol-free formaldehyde dissolved in phosphate buffered saline (PBS) washed and stored in blocking solution. Blocking solution was composed of 2% (v/v) normal goat serum (Gibco, Bornem, Belgium), 0.2% (w/v) powdered milk (Bio-Rad, Hercules, CA, USA), 1% (w/v) bovine serum albumin (BSA, Sigma-Aldrich, St. Louis, MO, USA), 0.1M glycine (Bio-Rad), 0.01% Triton X-100 (Bio-Rad), in PBS (Calbiochem, Darmstad, Germany) with 0.2% sodium azide (Sigma-Aldrich,). Oocytes and follicles were evaluated with laser scanning confocal microscopy (LSM170, ZEISS, Imager Z1) using a Zen 2008 imaging program, after being stained for anti-active-Caspase-3 (AC3, R&D systems, Minneapolis, USA), phospho-ataxia-telangiectasia-mutated (ATM, Millipore, Temecula, CA, USA) and γH2AX (BioLegend, San Diego, CA, USA). The percentage of granulosa cells from small preantral follicles positive for DSBs by γH2AX, apoptotic by AC3, and the ATM expression were compared to vehicle-treated controls. Vehicle-treated animals served as control. The extent of DSBs was further investigated by single cell gel electrophoresis (Comet assay) [6]. Briefly, GV oocytes were obtained and the granulosa cells were removed enzymatically by short incubation in hyaluronidase and the zona pellucida was removed by incubation in acid Tyrode's solution. Following the lysis of the oocytes with detergent at high salt concentration, electrophoresis was performed at neutral PH to facilitate the detection of DSBs (7.4). Chromatin staining was performed with propidium iodide. The extent of DNA migration was quantified using comet score program (TriTech Corporation, Sumerduck, VA, USA) and expressed as the percentage of DNA in the tail and compared with non-doxorubicin-treated oocytes. All the products used for this experiment were bought from Sigma-Aldrich, St. Louis, MO, USA, unless otherwise mentioned.

### Ovarian tissue histology and immunohistochemistry

OCT samples were recovered and processed for hematoxylin-eosin staining (H&E) and immunohistochemistry (IHC) with activated caspase-3 (AC3) antibodies for the detection of apoptosis. The number of apoptotic follicles was expressed as the fraction of total follicle numbers in each square millimeter of tissue. Tissues were also evaluated for the presence of new (CD31, Abcam, MA, USA) and existing mature blood vessels (alpha smooth muscle actin) (α-SMA, Ventana, Lille, France) by IHC staining. Blood vessels were counted and averaged at high power field magnification (×400) in a grid area of 0.15 mm^2^ at five randomly selected positions in twenty-five different sections of each tissue sample [5, 7]. The presence of characteristic nuclear foci after immunostaining for γH2AX (Bethyl Laboratory, Montgomery, TX, USA) was considered to represent the induction of DSBs and the percent of oocytes expressing distinct γH2AX foci was visually assessed under microscope.

### Statistical analysis

SPSS 17 for Windows package (SPSS Inc., Chicago, IL) was used for statistical analysis. To choose the appropriate statistical test, Levene's test of homogeneity of variances (p<0.01) and Kolmogorov-Smirnov test of normality (p<0.01) were performed. Continuous data (presented as mean±SD) were analyzed by one-way analysis of variance followed by the LSD post hoc test. The non-parametric data were analyzed with the Kruskal-Wallis test followed by multiple pair-wise comparisons using Mann-Whitney U-test (α value was adjusted). To analyze the relation between two categorical variables, Fisher's exact test was performed. Non-parametric correlation between the percentage of apoptotic follicles and the number of blood vessels was analyzed with the Spearman's test. The difference was considered statistically significant if the P value was <0.05.

## RESULTS

### Doxorubicin induces double strand DNA breaks in human ovary as reported by γH2AX expression

**Figure 1:**
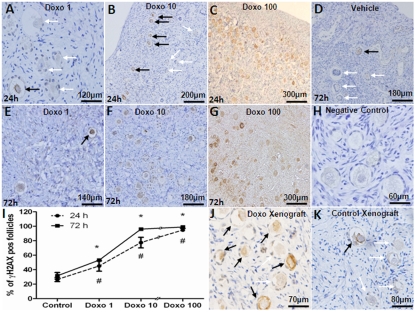
Induction of double strand DNA breaks in human primordial follicles by doxorubicin γH2AX-positive (black arrows) and negative (white arrows) primordial follicles in human ovarian cortical pieces after *in vitro* culture with doxorubicin doses of 1-100 mg/ml for 24 or 72h. A-C) Doxorubicin induces DSBs in a dose-dependent fashion after 24h of culture. Note the massive induction of DSBs in primordial follicles after 24h culture with doxorubicin 100 mg/ml. D) Vehicle control at 72h. E-G) Doxorubicin treatment at 100 mg/ml for 72h also causes ovarian tissue necrosis along with massive γH2AX expression in stromal cells. H) Negative control, secondary antibody omitted. I) Induction of DSBs was positively correlated with the dose of doxorubicin after 24-72h culture (Spearman's rank correlation coefficient: 0.967; p<0.001). J) High number of γH2AX-positive primordial follicles in a xenograft from a SCID mouse treated with 10mg/kg doxorubicin. K) Vehicle-treated (control) human ovarian tissue xenograft. # Significantly different from the control 24h * Significantly different from the control

For *in vitro* experiments, human ovarian cortical pieces were cultured for 24-72h with or without doxorubicin at 1, 10, 100 μg/ml doses and primordial follicle densities were compared. The presence of γH2AX, the reporter of DSBs [3, 4] and primordial follicle apoptosis, detected by the activated caspase-3, were studied in adjacent OCT sections. A significantly higher percentage of γH2AX-positive primordial follicles were observed in OCTs incubated with 1 μg/ml doxorubicin for 24h in comparison to vehicle-treated controls (45.2±7.3 vs. 26.5±2.9 %, p=0.005) and 72h (53.2±1.7 vs. 31.7±4.5 %, p<0.001) (Figure 1, A-H). Treatment with 10 μg/ml doxorubicin resulted in a significantly higher percentage of γH2AX-positive primordial follicles both after 24h (77.5±7.4 %, p<0.001) and 72h (96.2±7.2 %, p<0.001) compared to control. Doxorubicin 100 μg/ml induced massive γH2AX labeling in ovarian follicles regardless of the incubation time (94.8± 2.4 and 99.1±1.7 %, 24h and 72h respectively, p<0.001). A significant correlation was observed between the dose of doxorubicin and the percentage of γH2AX-foci positive primordial follicles in cultured OCTs (Spearman's rank correlation coefficient: 0.967; p<0.001) (Figure 1, I).

To translate the *in vitro* findings to the *in vivo* setting, SCID mice were xenografted with human ovarian tissue and received 10 mg/kg of doxorubicin as described in the methods section. Doxorubicin treatment resulted in a significantly higher percentage of γH2AX-positive primordial follicles compared to vehicle treatment (58.1±1.0 vs. 15.6±0.9 %, p<0.001) (Figure 1, J and K).

### Doxorubicin induces apoptotic death of human ovarian follicles

**Figure 2:**
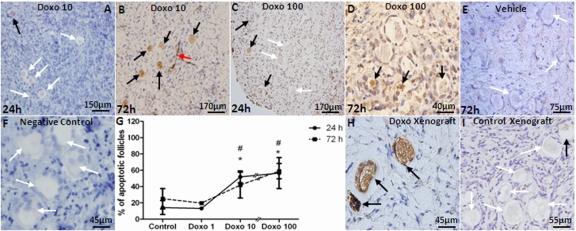
Induction of apoptotic primordial follicle death by doxorubicin AC3-positive (black arrows) and negative (white arrows) primordial follicles and apoptotic vasculature (red arrow) in human ovarian cortical pieces. A-D) Doxorubicin induces apoptosis of human primordial follicles after 24h culture in 10 and 100 μg/ml concentrations. E) Vehicle control at 72h. F) Negative control are shown where primary antibody omitted. G) A significant correlation was observed between the dose of doxorubicin and percentage of apoptotic primordial follicles in cultured OCTs (Spearman's rank correlation coefficient: 0.785; p=0.0001). H) A high number of apoptotic primordial follicles was seen in human ovarian tissue xenografted into the dorsal muscle of SCID mouse after treatment with 10mg/kg doxorubicin. I) Xenogafted vehicle-treated control. * Significantly different from controls at 24 or 72h. # Significantly different from 1 μg/ml doxorubicin dose at 24 and 72h.

Doxorubicin caused apoptosis in a dose-dependent manner when evaluated by AC3 staining *in vitro* (Figure 2, A-F). The percentage of follicles undergoing apoptosis increased at both 10 and 100 μg/ml but statistical significance was noted only with the 100 μg/ml dose of doxorubicin after 24 and 72h of culture (57 and 59 % of follicles in doxorubicin-treated vs. 14 and 25 % in vehicle-control at 24h and 72h, respectively, p<0.001). A significant correlation was observed between the dose of doxorubicin and the percentage of apoptotic primordial follicles in cultured OCTs (Spearman's rank correlation coefficient: 0.785; p=0.0001)(Figure 2, G).

**Figure 3:**
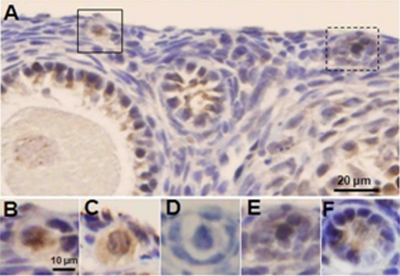
Induction of DSBs and apoptotic follicle death by doxorubicin in xenografted SCID mouse ovaries Ovaries of xenografted SCID mice were recovered 24h after a single injection of doxorubicin 10 mg/kg and stained for γH2AX and AC3. A) γH2AX-positive primordial follicle oocytes (highlighted) in lower magnification. Note γH2AX staining in granulosa cells but not the oocyte of the adjacent preantral follicle. B) Higher magnification of the primordial follicle in solid square with yH2AX staining. C) The same primordial follicle in B shows AC3-positive staining on the adjacent section, suggesting that DSBs induced apoptotic follicle death. D) Negative control. Primary antibody omitted, same follicle as in B & C, on adjacent section, E) Higher magnification of γH2AX-positive primordial follicle from panel A, in dashed square. F) AC3-positive staining of the same follicle in D on adjacent section.

Consistent with *in vitro* findings, *in vivo* doxorubicin treatment induced a significantly higher level of apoptosis in xenografted primordial follicles (55.4±5.7 vs. 11.8±5.7 %, p<0.005) treated with doxorubicin compared to vehicle-treated controls (Figure 2, H and I). In addition, the ovaries of SCID mice that were used in the human xenografting experiments showed that doxorubicin treatment significantly reduced the mean density of primordial follicles (4.31±2.1 vs. 10.57±6.7/mm^2^, p<0.001) compared to vehicle-treated controls similar to its effect in human ovarian xenografts in the same mice, doxorubicin treatment also induced a significantly higher percentage of apoptotic follicles in primordial (47.1±12.8 vs. 0.4±1.4 %, p<0.001), primary (44.7±12.2 vs. 4.1±5.9 %, p<0.001) and developing (49.5±8.6 vs. 6.9±5.5 %, p<0.001) follicles as well as inducing γH2AX expression (Figure 3). In these follicles, co-expression of AC-3 and γH2AX suggested that unrepaired DSBs were resulting in apoptotic death.

### Doxorubicin causes microvascular and stromal damage in human ovary

**Figure 4:**
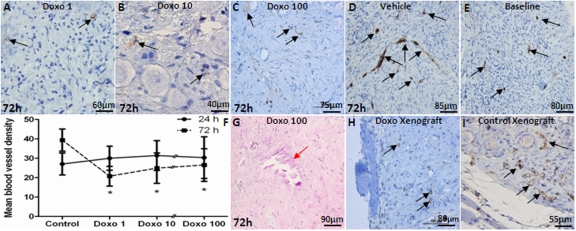
The impact of doxorubicin on vascular density in ovarian cortical pieces A-C) CD31-positive blood vessels in human ovarian cortical pieces cultured with different doses of doxorubicin for 72h. Doxorubicin treatment results in decreased density of blood vessels in a dose dependent manner. Vehicle-treated control. E) CD31-positive blood vessels are shown at baseline in ovarian tissue. F) Doxorubicin caused a significant decrease in ovarian vascular density after 72h for all doses but not 24h culture compared to controls at all three doses tested. G) Doxorubicin-induced necrosis and stromal cell death *in vitro* (red arrow). H&I) Similar to *in vitro* experiments, doxorubicin reduced vascular density in xenografts compared to vehicle-treated control xengrafts. Black arrows show CD31-positive blood vessels in human ovarian cortical pieces. * Significantly different from the control (72h).

The density of newly forming ovarian microvessels was determined by CD31-expression in micro vessels per mm^2^ after treatment with doxorubicin. The results showed a decrease in vascular density with the 72h culture after 1 μg/ml (26.5±8.4/mm^2^, p<0.001), 10 μg/ml (25.5±7.8/mm^2^, p<0.05), and 100 μg/ml doxorubicin (20.8±5.1/mm^2^, p<0.05) compared to vehicle treatment (39.4 ±5.7/mm^2^)(Figure 4, A-D). In contrast, blood vessel density increased significantly after 72h culture in vehicle-treated controls compared to the uncultured baseline tissue (39.4±5.7/mm^2^ vs. 30.0±6.2/mm^2^, p<0.001), (Figure 4, D and E), due to expected continuation of microvessel proliferation. There was also a significant decrease in the density of mature blood vessels as determined by αSMA IHC after 72h culture with 10 μg/ml (4.0±2.0 vs. 9.5±3.5, p=0.002) and 100 μg/ml doxorubicin (4.7±3.1 vs. 9.5±3.5, p=0.04) compared to controls. A significant inverse-correlation was observed between the dose of doxorubicin and the density of αSMA-positive vessels (Spearman's rank correlation coefficient: −0.798; p<0.001)(Figure 4, F). Furthermore, after 72h culture with doxorubicin 1, 10 and 100 μg/ml, multiple necrotic areas were seen in OCTs (7.3±5.1, mean number of necrotic areas/graft)(Figure 4, G). Validating and confirming *in vitro* organ culture experiments, doxorubicin treatment also resulted in reduced vascular density in xenografted human ovarian tissues compared to vehicle-treated controls (6.4±1.9 vs. 13.9±2.1, p<0.001)(Figure 4, H and I).

### Doxorubicin induces γH2AX in immature (GV) mice oocytes and small ovarian preantral follicles, while activating ATM-pathway response

**Figure 5:**
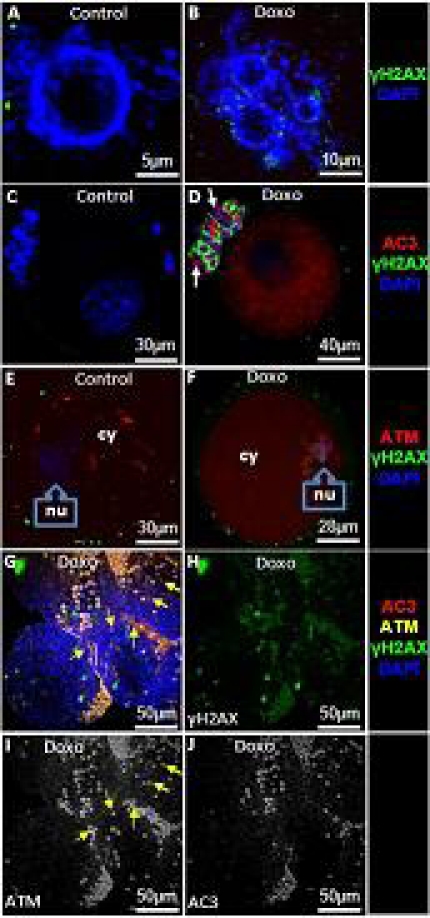
Doxorubicin induces double strand DNA breaks, activates ATM, and causes apoptosis in mouse oocytes To study the *in vivo* effect of doxorubicin on mouse ovarian follicles and their cumulus cells, animals were injected with a single dose of doxorubicin 10 mg/kg. GV oocytes and small preantral follicles were retrieved 24h later from ovaries and evaluated by confocal microscopy. A&B) Doxorubicin treatment leads to multinucleation as well as increased expression of γH2AX. C&D) Doxorubicin-induces apoptosis of the oocyte and surrounding granulosa cells (arrows) in association with the induction of DSBs. E&F) Expression and localization of ATM in oocytes after doxorubicin treatment. While a higher amount of ATM is localized in the cytoplasm (cy) of the control oocytes (E), doxorubicin-treated oocytes show higher localization in the nucleus(nu)(F). G) Consistent with its impact on oocytes, ATM expression also increases in granulosa cells of small antral follicles after doxorubicin treatment (yellow arrows). H) Note that doxorubicin treatment induces DSBs in granulosa cells of small antral follicles (Split channel from image G). I) A minority of granulosa cells are non-apoptotic and express ATM indicating that DNA repair might have enabled them to overcome genotoxic stress (yellow arrows) (split channel from image G). J) The majority experience apoptosis despite the activation of ATM (split channel from image G).

To further delineate and confirm the impact of doxorubicin on double strand DNA and the involvement of ATM-mediated DSB repair response, GV oocytes and small preantral follicles were collected from the ovaries of doxorubicin- or vehicle-treated mice and evaluated by confocal microscopy. A significantly higher number of GV oocytes in doxorubicin-treated animals demonstrated multi-nucleation compared to vehicle-treated controls (28.2±6.0 vs. 4.9±4.0, p <0.001) (Figure 5, A and B) consistent with chromatin damage and genotoxicity [8, 9]. In parallel with multi-nucleation, the mean number of γH2AX foci (64.1±29.8 vs. 11.5±7.3, p<0.05) and the percentage of apoptotic follicles (42.7±11.6 vs. 2.7±3.8, p<0.05) were significantly higher in GV oocytes from doxorubicin-treated ovaries compared to vehicle-treated controls (Figure 5, C and D).

**Figure 6:**
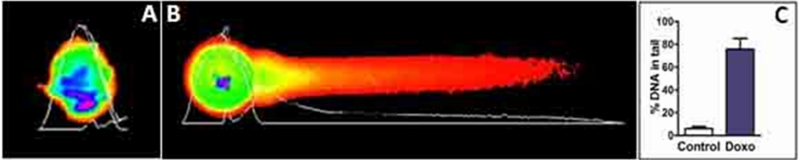
Comet assay with murine GV oocytes Double strand DNA breaks were detected in murine GV oocytes using a single cell gel electrophoresis (Comet assay) after 24h culture with or without doxorubicin (10 μg/ml). Representative images of non-treated (A) and doxorubicin-treated (B) oocytes are shown. (C) Quantification of DNA damage in control versus doxorubicin treated oocytes; expressed as mean (± SD) % of DNA in the tail. P = 0.002 (t-test).

The *comet* assay is based on the ability of negatively charged fragments of DNA to move through an agarose gel in response to an electric field. The extent of DNA migration depends directly on the DNA damage present in the cells. The alkaline version of the *comet* assay enables the detection of DNA single-strand breaks and alkali-labile lesions (DNA breaks that only become apparent after exposure to alkali) while electrophoresis at neutral conditions detects DSBs[6]. To confirm that increased γH2AX foci reflected DSBs, we performed a neutral pH comet assay on GV oocytes treated *in vitro* with doxorubicin 10 μg/ml. for 24h and found similar results (Fig 6).

Having shown that doxorubicin causes DSBs resulting in apoptotic death in oocytes, we next studied the DNA repair response. To study the DNA repair response, we analyzed expression of activated ATM. Compared to vehicle-treated controls, doxorubicin treatment increased ATM activation both in the nucleus (7.1±1.4 vs. 224.5±42.2, p<0.001) and the cytoplasm (142.2±30.1 vs. 186.3±27.6, p<0.05) (Figure 5, E and F). Moreover, the same treatment caused a change in the localization of ATM. While in control oocytes the predominant fraction of ATM was in the cytoplasm (142.2±30. vs. 7.1±1.4 in nucleus, p<0.001) after doxorubicin treatment (Figure 5, E), a larger proportion was located in the nucleus (224.5±42.2 vs. 186.3±27.6 in cytoplasm, p<0.05)(Figure 5, F). The latter indicates a significant translocation of phospho-ATM from cytoplasm to nucleus.

Unrepaired DSBs will result in the activation of apoptotic pathways via the ATM-pathway [10] or cell cycle arrest [11]. We therefore assessed the relationship between γH2AX staining and apoptotic oocyte death. Of the γH2AX-positive oocytes, 66.3 ±12.8% were apoptotic and the remainder were non-apoptotic based on AC-3 staining. This suggests that either the DNA damage was not sufficient to induce cell death, or DNA repair mechanisms were successful and prevented cell demise.

To demonstrate that ATM activation was involved in and is related to oocyte survival or apoptotic death after doxorubicin-induced DNA damage, we pretreated mouse GV oocytes with ATM inhibitor KU-55933, 10 μMol (Tocris Bioscience, Missouri, USA) or its vehicle (dimethyl sulfoxide, Sigma) for 3h prior to doxorubicin treatment (1 μg/ml). Oocyte apoptosis was assessed 24h later. In the ATM-inhibited group, percentage of apoptotic oocytes was significantly reduced compared to the non-ATM-inhibited (28.6% vs. 64.1%; p<0.05). These data indicate that ATM activation is involved in chemotherapy-induced oocyte death and is consistent with the previous data in lymphoblastoid cells that inhibition of ATM rescues from *tumor necrosis factor- related apoptosis-inducing ligand* induced apoptotic death [12]. Interestingly, doxo treatment blocked cell cycle progression beyond Metaphase I (MI) stage but ATM inhibitor treatment did not significantly affect this inhibition. One hundred percent in the doxo group and 92.1% in the doxorubicin+ATM inhibitor group arrested at GV or MI compared to 17.7% in the control group after 24h in culture (P<0.001).

TAp63α is involved in female germline survival and ATM has been shown to regulate c-Abl-TAp63 pathway[13, 14, 15]. Thus we wanted to determine whether the reduction of doxo-induced apoptotic death of oocytes in response to the ATM inhibitor was due to prevention of ATM-induced activation of the c-Abl-TAp63 pathway. By confocal imaging, we found that ATM inhibition with KU-55933 also inhibited TAp63 α and c-Abl activation in the nuclei of doxo-treated oocytes (supplementary figure 1). The inhibition of TAp63 was more profound, with KU-55933 completely blocking phosphorylation of TAp63 in response to genotoxic effects of doxorubicin. This suggests that ATM is regulating TAp63 α activation both c-Abl dependent and independent pathways, the latter being the predominant form.

Doxorubicin treatment also resulted in a significant rise in the percentage of apoptotic cumulus cells surrounding the oocytes compared to controls (27.7±10.3 vs. 2.4±0.6, p<0.001) (Figure 5, C and D). Thus we also studied cell survival and DNA damage in granulosa cells of small follicles isolated from doxorubicin-treated mice. Consistent with its effect in GV oocytes, doxorubicin compared to vehicle treatments resulted in a significant increase of cells expressing γH2AX (53.8±12.3 vs. 2.1±0.4, p<0.001) and induction of apoptosis in granulosa cells (35.9±14.6% vs. 3.7±2.0%, p<0.001)(Figure 5, G-J). In doxorubicin-treated follicles higher percentage of granulosa cells expressed activated ATM (57.5±17.8 vs. 0.9± 0.7, p<0.001). A fraction of granulosa cells that co-expressed ATM and γH2AX were not apoptotic (12.1± 5.9%) suggesting that ATM-mediated repair process was still ongoing and probably preventing cell death (Figure 5, I). In majority however, apoptosis coincided with the co-expression of ATM and γH2AX (Figure 5, J), indicating that the DNA repair response was not sufficient to rescue granulosa cells from chemotherapy-induced death.

## DISCUSSION

Doxorubicin is a key chemotherapeutic agent used in the treatment of numerous malignancies such as breast, ovarian, and endometrial cancers as well as lymphomas, acute leukemias, and many others which are commonly encountered during the premenopausal ages [16, 17, 18, 19]. In the present study we investigated the mechanism of chemotherapy-induced ovarian aging induced by this drug *in vitro* and *in vivo* on human and mice ovaries.

We found that doxorubicin, in a dose-dependent fashion, caused massive induction of γH2AX, likely reporting formation of DSBs in human and mouse oocytes, as confirmed by the presence of multi-nucleation (Fig. 5 A&B) and comet assay (Supplementary figure 1). The induction of DSBs was associated with apoptotic death of primordial follicles upon exposure to doxorubicin. Doxorubicin activated ATM-mediated DSB repair pathways, which involved H2AX phosphorylation and most likely activation of other signaling pathways associated with DNA damage response [4]. It seems that following DNA damage not only expression of activated ATM was increased but there was a significant translocation from the cytoplasm to the nucleus. Although ATM was originally thought to be a nuclear protein in proliferating cells, in oocytes it is predominantly cytoplasmic [20]. To our knowledge, this is the first observation of activated ATM behavior in oocytes. Further laboratory research will be needed to determine the function of cytoplasmic ATM and the significance of the translocation process to ATM function in response to genotoxic stress.

It is clear that the extent of doxorubicin-induced DNA damage is sufficient to induce apoptotic death of the majority of human and mouse primordial follicles. Apparently the repair mechanisms were inadequate to rescue them. Our findings, however, also suggest an intriguing possibility that the activation of ATM may result in the rescue of a considerable minority of oocytes which have sustained DSBs due to chemotherapy. While the ATM-mediated DSB repair pathway is of high fidelity in nature and the majority of oocytes which were made to survive by this mechanism should have their DNA integrity restored, miss-repair or survival despite a failed repair is still possible[21]. This novel concept is supported by our demonstration of reduction of chemotherapy-induced apoptotic death after the treatment of oocytes with ATM inhibitor KU-55933. While in somatic cells, inhibition of DNA repair mechanisms may be expected to increase sensitivity to chemotherapeutics in general [22], germ cells seem to have a different behavior. In oocytes, TAp63α, a pro-apoptotic P53 family member with specificity to germ line, is activated in response to DNA damage and causes apoptotic death [13, 15]. TAp63α is activated by c-Abl, which is a non-receptor tyrosine kinase involved in many cellular processes, in response to DNA damage. It has been shown that ATM is involved in activation of c-Abl after DNA damage [14]. Our finding that ATM inhibition reduces apoptotic oocyte death after DNA damage, while blocking TAp63α and c-Abl activation, is consistent with ATM's role in activation of the c-Abl-TAp63 pathway. We found that ATM inhibition had a more profound negative effect on Tap63α activation than c-Abl (Suppl. Fig. 1), suggesting that ATM might also be regulating doxo-induced TAp63α activation independent of c-Abl in oocytes. Our findings are also in parallel with the data showing that ATM has a pro-apoptotic function in DNA-damaged neurons of developing mouse CNS, in collaboration with the Bax protein [23, 24, 25, 26, 27]. It was observed in these previous studies that ATM−/− mice exhibited resistance to ionizing radiation-induced apoptosis in diverse regions of the developing CNS. In wild-type but not ATM−/− mice, up-regulation of p53 coincided with cell death, suggesting that ATM-dependent apoptosis in the CNS was mediated by p53. Furthermore, p53-null animals exhibited similar lack of radiation-induced cell death in the developing nervous system. These results are in correlation with our findings in the ovary, and suggest that ATM may function at a developmental survival checkpoint that serves to eliminate cells with excessive DNA damage[23, 25, 26] [27, 28].

This brings the next question of whether unrepaired or mis-repaired DSBs can culminate into severe mutagenic changes in the surviving primordial follicles. Even though clinical data do not suggest an increase in the incidence of genetic disorders in children born to cancer survivors, the power of such studies is limited [29, 30]. Of concern, a recent rodent study showed increased rates of neonatal death, physical malformations, and chromosomal abnormalities in up to 6 generations of offspring after exposure to single dose of doxorubicin [31]. Because unrepaired DSBs can result in severe mutagenesis and can explain these new findings in laboratory animals, it will be of great importance to study the accumulation of DSBs in long term human ovarian xenografting experiments as well as *in vivo* in mice. However, we found from our *in vitro* experiments with mouse oocytes that even though ATM inhibition rescued oocytes from chemotherapy-induced death, it did not prevent the arrest of their growth at M-I stage. This indicates that there are cell cycle check mechanisms independent of ATM which can sense lethal DNA damage and prevent propagation of severe mutations. It is possible that in humans too, even though some primordial follicles may survive chemotherapy exposure despite the presence of significant DSBs, their growth may be halted after ovulation triggers the resumption of cell cycle from prophase-I. Because cell cycle check mechanisms would prevent such oocytes from becoming mature and advancing to M-II stage, they cannot be fertilized to become embryos. This hypothesis is consistent with our previous clinical findings that women with seemingly normal ovarian reserve post-chemotherapy have significantly lower pregnancy rates with *in vitro* fertilization compared to age-matched controls [32]. Hence women whose ovarian function has survived chemotherapy may still be at risk for infertility due to this occult DNA damage in their remaining oocytes.

Why some ovarian follicles die after chemotherapy while others survive in the same environment? It is possible that some oocytes may survive chemotherapy without DNA damage due to their individual resistance to chemotherapy through yet undefined mechanisms. One such possible mechanism is the difference in the efficiency of DNA repair in individual oocytes. Data are already accumulating that effective DSB repair is vital for oocyte survival [33]. It is possible that those oocytes which inherently are more efficient in DNA repair are more likely to be resistant to chemotherapy-induced DSBs and cell death. The opposite is also possible that the absence of ATM response is responsible for the perceived survival of ovarian follicles after chemotherapy. From our experiments showing that ATM inhibition reduces apoptotic oocyte death, it can be speculated that some oocytes that survive despite chemotherapy exposure might have deficient ATM response, which fails to initiate TAp63α dependent death, allowing survival despite the persistence of DSBs. If such individual differences exist, exploration of factors regulating DSB repair in oocytes may result in the development of new therapeutic strategies to protect oocytes by enhancing DNA repair efficiency and preserve fertility in the face of chemotherapy.

It should be noted the survival of primordial follicles was presently observed at relatively short time (24–72 h) of exposure to chemotherapy in organ cultures or xenografts (figs 2 and 3). It is possible that some of the “surviving” oocytes were actually undergoing cellular senescence, the state of reproductive cell death with preservation of physical and metabolic integrity [34]. In fact, the mechanism of chemotherapy-induced growth inhibition of tumors often involves both, induction of apoptosis and irreversible impairment of cell reproductive capacity, defined as “senescence-like growth arrest”, “premature senescence” or “drug-induced senescence” [34, 35, 36]. Further research will be needed to determine whether meiotic cells *i.e.* oocytes can also experience this form of senescence.

Another novel finding from our studies is the ability of doxorubicin to damage human ovarian microvasculature. We found that doxorubicin causes a decline in microvascular density in a dose-dependent fashion. While we cannot prove that microvascular damage directly contributes to chemotherapy-induced follicle death, in prior studies we showed an inverse correlation between human ovarian microvascular density and primordial follicle apoptosis[5]. It is generally believed that the human primordial follicles are not acutely dependent on perfusion but the clinical experience from ovarian transplantation[37] as well as acute ovarian ischemia (ovarian torsion) cases do not support this general belief[38]. While larger follicles may be more sensitive to acute ischemic changes due to a greater mass of proliferating granulosa cells, our experimental models clearly support the dependence of primordial follicles on adequate vascularization [5]. Given this background, it is possible that chemotherapy-induced ovarian vascular damage is a contributing factor to the loss of primordial follicle reserve and aging in human ovary.

Furthermore, ovarian tissue is a functional endocrine organ even after natural menopause or premature ovarian failure, and continues to produce critical sex steroids such as testosterone and estrogen. We not only showed that doxorubicin-induces microvascular damage but it also damages ovarian stroma. Hence it is likely that even as a postmenopausal senescent organ, the human ovary becomes dysfunctional after chemotherapy. In keeping with this concept, we previously showed that chemotherapy exposed ovarian stromal tissue produces lower levels of estradiol compared to controls [39].

Our findings have strong clinical implications. A large number of young females receive doxorubicin as part of their treatment for cancer. Doxorubicin treatment may not only be compromising the future fertility potential and accelerating ovarian aging in these young cancer survivors but can also be placing them at disadvantage during their postmenopausal years. Women undergoing chemotherapy with doxorubicin containing regimens must undergo adequate counseling to weigh their options for established and experimental fertility preservation options [40](9). While oocyte and embryo cryopreservation can be utilized to preserve future fertility, ovarian cryopreservation for future transplantation is the only modality that can restore natural ovarian function and circumvent chemotherapy-induced premature ovarian senescence [41, 42]. Given our findings that the chemotherapy-exposed human ovary may not function as a normal postmenopausal organ and show accelerated senescence, ovarian tissue freezing may be selectively attractive to some survivors. More importantly, given these complex damaging effects of cancer drugs on human ovarian function, less toxic drugs should be developed, or elected when they are available. And finally, understanding the mechanism of accelerated senescence by cancer drugs may enable us to develop targeted pharmacological ovarian preservation methods.

## FUNDING

This study was supported by the National Institute of Child Health and Development as well as National Cancer Institute, National Institute of Health (HD 053112 and R21HD061259) to KO. In addition, ZD is supported by RO1 NCI 28 704.

## SUPPLEMENTAL MATERIAL



## Abbreviation

α-SMA: Alpha Smooth Muscle Actin
ATM: Ataxia Telangiectasia Mutated
Cy: Cytoplasm
DSB: Double Strand DNA Bbreaks
H&E: Haematoxylin Eeosin
IHC: Immunohistochemistry
MI: Metaphase I
Nu: Nucleus
OCT: Ovarian Cortical Tissue
SCID: Severe Combined Immunodeficiency
