# Supplementary material for: Mechanisms of chemotherapy-induced human ovarian aging: double strand DNA breaks and microvascular compromise
Source: Aging (Albany NY). 2011 Aug 22;3(8):782–93. doi: 10.18632/aging.100363 (PMC3184979; doi:10.18632/aging.100363)
Supplement: Supplementary file 1 [file aging-03-782-s001.pdf]

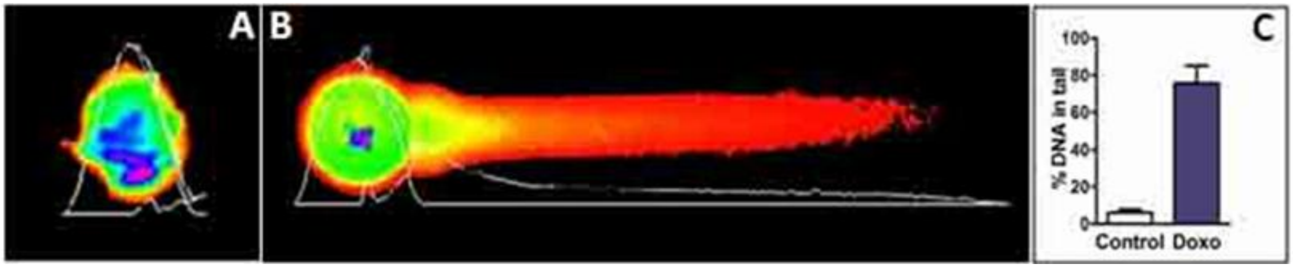

Figure 6. Comet assay with murine GV oocytes. Double strand DNA breaks were detected in murine GV oocytes using a single cell gel electrophoresis (Comet assay) after 24h culture with or without doxorubicin (10 µg/ml). Representative images of non-treated (A) and doxorubicin-treated (B) oocytes are shown. (C) Quantification of DNA damage in control versus doxorubicin treated oocytes; expressed as mean (± SD) % of DNA in the tail. P = 0.002 (t-test).

expressed ATM and γH2AX were not apoptotic (12.1± 5.9%) suggesting that ATM-mediated repair process was still ongoing and probably preventing cell death (Figure 5, I). In majority however, apoptosis coincided with the co-expression of ATM and γH2AX (Figure 5, J), indicating that the DNA repair response was not sufficient to rescue granulosa cells from chemotherapy-induced death.

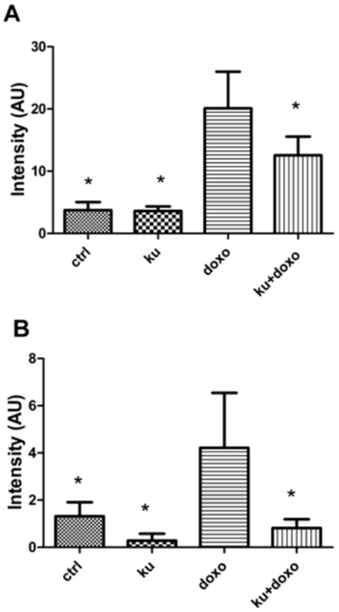

Supplementary Figure 1. ATM inhibitor KU-55933 blocks activation of p63 in mouse oocytes response to genotoxic stress. Germinal vesicle stage oocytes from 6 week old FVB mice (n=14-16/group) were treated without or with doxorubicin (1 µg/ml) for 24h in the presence or absence of KU-55933 (10 µMol) and were subjected to confocal microscopy. Signal intensity for phospho-c-Abl (A) and phospho-TAp63 (B) were quantified using ImajeJ software (arbitrary units, AU). (A) KU-55933 inhibits doxo-induced activation of c-Abl ($p<0.05$) but not the basal levels of phospho-c-Abl; (B) KU-55933 completely blocks activation of p63 ($p<0.05$) both in non doxo-treated ($p<0.05$) and doxo-treated ($p<0.05$) oocytes. * indicates statistically significant difference from the doxo only group.

DISCUSSION

Doxorubicin is a key chemotherapeutic agent used in the treatment of numerous malignancies such as breast, ovarian, and endometrial cancers as well as lymphomas, acute leukemias, and many others which are commonly encountered during the premenopausal ages [16,17,18,19]. In the present study we investigated the mechanism of chemotherapy-induced ovarian aging induced by this drug *in vitro* and *in vivo* on human and mice ovaries.

We found that doxorubicin, in a dose-dependent fashion, caused massive induction of γH2AX, likely reporting formation of DSBs in human and mouse oocytes, as confirmed by the presence of multi-nucleation (Fig. 5 A&B) and comet assay (Supplementary figure 1). The induction of DSBs was associated with apoptotic death of primordial follicles upon exposure to doxorubicin. Doxorubicin activated ATM-mediated DSB repair pathways, which involved H2AX phosphorylation and most likely activation of other signaling pathways associated with DNA damage response [4]. It seems that following DNA damage not only expression of activated ATM was increased but there was a significant translocation from the cytoplasm to the nucleus. Although ATM was originally thought to be a nuclear protein in proliferating cells, in oocytes it is predominantly cytoplasmic [20]. To our knowledge, this is the first observation of activated ATM behavior in oocytes. Further laboratory research will be needed to determine the function of cytoplasmic ATM and the significance of the translocation process to ATM function in response to genotoxic stress.

It is clear that the extent of doxorubicin-induced DNA damage is sufficient to induce apoptotic death of the majority of human and mouse primordial follicles. Apparently the repair mechanisms were inadequate to
